# CDK Blockade Using AT7519 Suppresses Acute Myeloid Leukemia Cell Survival through the Inhibition of Autophagy and Intensifies the Anti-leukemic Effect of Arsenic Trioxide

**DOI:** 10.22037/ijpr.2019.112560.13827

**Published:** 2019

**Authors:** Mitra Zabihi, Ava Safaroghli-Azar, Ahmad Gharehbaghian, Mehdi Allahbakhshian Farsani, Davood Bashash

**Affiliations:** a *Department of Hematology and Blood Banking, School of Allied Medical Sciences, Shahid Beheshti University of Medical Sciences, Tehran, Iran. *; b *Student Research Committee, Department of Hematology and Blood Banking, School of Allied Medical Sciences, Shahid Beheshti University of Medical Sciences, Tehran, Iran.*

**Keywords:** Acute myeloid leukemia (AML), KG-1 cells, Cyclin-dependent kinase (CDK), AT7519, Autophagy, Arsenic trioxide (ATO)

## Abstract

The strong storyline behind the critical role of cyclin-dependent kinase (CDK) inhibitor proteins in natural defense against malignant transformation not only represents a heroic perspective for these proteins, but also provides a bright future for the application of small molecule inhibitors of CDKs in the novel cancer treatment strategies. The results of the present study revealed that the inhibition of CDKs using pan-CDK inhibitor AT7519, as revealed by the induction of G1 cell cycle arrest as well as the reduction of cyclins expression, resulted in decreased survival in acute myeloid leukemia (AML)-derived KG-1 cells, either in the context of single agent or in combination with arsenic trioxide (ATO). Apart from alterations in the expression of proliferation and apoptotic genes, the anti-survival property of AT7519 was coupled with the inhibition of autophagy-related genes. Notably, we found that the blockage of autophagy system in KG-1 cells resulted in a superior cytotoxic effect, introducing autophagy as a probable suppressor of cell death. As far as we are aware, to date, no study has reported the contributory mechanisms correlated with the less sensitivity of acute leukemia cells to AT7519 and our study suggested for the first time that the activation of both PI3K and c-Myc signaling pathways could overshadow, at least partly, the efficacy of this agent in KG-1 cells. Overall, due to the pharmacologic safety of AT7519, our study proposed this inhibitor as a promising agent for the treatment of AML either as a single agent or in a combined-modal strategy.

## Introduction

Being the most critical enzymes for controlling the progression of the cell cycle, cyclin dependent kinases (CDKs) are considered as accessible weakening point to be exploited by malignant cells to provide a platform for unrestricted cell proliferation (1). Through bifurcating at many points, these groups of specific serine/threonine kinases take place in the regulation of diverse intracellular functions and play a dominant role in the stimulating oncogenic signals leading to cancer cell immortalization (2). As the results of mounting body of studies found that CDKs are frequently over-expressed in human leukemia and also suggested that these enzymes have a crucial impact on the cancer cells sensitivity to common chemotherapeutic drugs (3), intensive focus has been allocated to find a suitable drug capable of CDKs suppression (4, 5). On the other hand, identification of the interaction between CDKs and oncogenic pathways has also provided more depth on this superfamily, suggesting that an expanding repertoire of novel drugs targeting CDKs could be beneficial therapeutic agents to be incorporated into the management of human leukemia (6). In this setting, new candidates have joined the panoply of drugs specifically targeting CDKs, which among them AT7519 was the first in class which entered into the highest phases of the clinical trials. 

In contrast to the first generation counterparts with the ability of targeting only CDK2 (7), AT7519 is able to inhibit multiple CDKs giving it a potent efficacy to block cell proliferation at different aspects (8). This unique characteristic has donated a remarkable anti-cancer property to this small molecule inhibitor and its efficient cytotoxic effect has been reported in a wide variety of human cancers ranging from solid tumors (9, 10) to hematologic malignancies (11, 12). Despite its prodigious effect in *in-vitro* experiments, AT7519 exerted a broad-spectrum antitumor activity with the favorable pharmacokinetic profile in xenograft models (13). It has been reported that AT7519 could repress tumor growth in both 5-FU-resistant colon cancer xenograft and paclitaxel-resistant cervical cancer xenograft (14). It has been also suggested that AT7519 could be an effective combinational agent that may inhibit progression of cervical cancer (10). The excellent characteristics of this agent became even more highlighted when the results of a clinical trial in non-Hodgkin’s lymphoma declared a safe dosage for AT7519 at which the plasma concentrations of the inhibitor was above the biologically active concentrations, meanwhile, it did not evolve significant adverse effects (14). Even though current studies mostly put AT7519 under the magnifying glass to evaluate its anti-cancer property as well as its safety profile, little is known about its mechanism of action. As far as we are aware, to date, no study has reported the contributory mechanisms correlated with the less sensitivity of acute leukemia cells to AT7519 and our study suggested for the first time that the activation of both PI3K and c-Myc signaling pathways could overshadow the efficacy of this agent in AML-derived KG-1 cells. Moreover, we found that the suppression of CDKs in an innovative combined-modality strategy decreased the survival of arsenic trioxide-treated KG-1 cells, while reducing the toxic concentrations of this chemotherapeutic drug. 

## Experimental

*Chemicals*

The small molecule inhibitors AT7519 (pan-CDK inhibitor), 10058-F4 (c-Myc inhibitor), CAL-101 (selective-p110δ inhibitor), and Bortezomib (proteasome inhibitor) were purchased from Selleckchem (Munich, Germany). To prepare a stock solution, all agents were dissolved in dimethylsulfoxide (DMSO) and then were divided to aliquots, and stored at −20 °C until use. Moreover, we used arsenic trioxide (ATO) (SinaDaroo) and autophagy inhibitor chloroquine (Sigma-Aldrich, Germany) for further experiments. 

*Cell culture and reagents*

To investigate the impact of CDK inhibition on acute myeloid leukemia, KG-1 cell line was chosen. For drug treatment, cells were grown in suspension in RPMI 1640 medium supplemented with 2 mM l-glutamine, 10% heat inactivated fetal bovine serum in humidified incubator. KG-1 cells were also treated with the relevant amounts of the anti-cancer agents and equal amounts of solvents, as an alternative control at the final concentration of 0.1%.

*Assessment of cell distribution in the cell cycle using flowcytometry*

The impact of AT7519 on the progression of the cell cycle was analyzed by PI staining. After treatment of KG-1 cells with the designated concentrations of AT7519 up to 48 h, cells were harvested, washed twice with cold PBS, and then fixed in 70% ethanol overnight. Afterwards, for DNA staining and RNA degradation, we added PI and RNase, respectively. Cells were then incubated for further 30 min and the distribution of cells was evaluated by flow cytometry. The final data was interpreted using the Windows FlowJo v.10 software.

*Trypan blue exclusion assay*

Trypan blue assay was tested on 150 × 10^3 ^KG-1 cells seeded in 24-well plate in the medium containing increasing concentrations of AT7519, either as a single agent or in combination with other anti-cancer agents. Following indicated time intervals, the centrifuged cells pellets were re-suspended in serum-free complete medium and then an equivalent amount of 0.4% trypan blue was added. The numbers of viable cells were counted manually and the percentage of viability was assessed.

*Detection of metabolic activity by micro-culture tetrazolium test*

KG-1 cells (5 × 10^3^) were seeded in 96-well plate in the presence of increasing concentrations of the agents and kept in a humidified 5% CO_2_ incubator at 37 °C. At different time intervals, the relevant amount of MTT solution (5 mg/mL in PBS) was added to each well and incubated at 37 °C for 3 h. The percentage of the metabolic activity of cells were evaluated by dividing the optical densitometry (OD) of a resulting formazan measured by an enzyme-linked immunosorbent assay (ELISA) reader in the drug-treated group by the control group. 

*Assessment of DNA synthesis rate*

To investigate whether CDK suppression is coupled with the reduction of DNA synthesis rate, bromodeoxyuridine (BrdU) based cell proliferation ELISA kit (Roche Molecular Biochemicals, Mannheim, Germany) was applied. Cells were cultured with different concentrations of AT7519 and 12 h prior to its incubation time ends, 10 μL of BrdU solution was added. Then cells were fixed and denatured by FixDenat solution. Following the addition of anti-BrdU antibody conjugated with peroxidase and etramethylbenzidine the reaction product was quantified by measuring the absorbance at 450 nm.

*RNA extraction, cDNA synthesis and quantitative real-time PCR*

Total RNA from KG-1 cells was extracted using RNA Isolation Kit (Roche, Mannheim, Germany) and quantified by Nanodrop instrument. The reverse transcription reaction was performed using Complementary DNA (cDNA) Synthesis Kit (Takara Bio, Shiga, Japan). Complementary DNAs (cDNAs) was subjected to quantitative real-time PCR (qRT-PCR) and then, fold change values were calculated based on 2^−ΔΔCt^ relative expression formula. 

*Detection of apoptosis using flowcytometry*

To explore the effect of AT7519 on induction of programmed cell death, cells were subjected to apoptosis analysis. AT7519-treated cells were harvested after both 24 h and 48 h of treatment, and after adding annexin-V-Flous and incubation for 20 min, fluorescence intensity was measured using flow cytometry. The experiment process has been described in detail in our previous article (15). 

*Determination of combination index and dose reduction index*

To investigate efficacy of drug combinations, the reduction of cell survival was examined by MTT assay and the combination index (CI) and dose reduction index (DRI) were evaluated by CalcuSyn software developed by Chou and Talalay. CI was calculated according to the classic isobologram equation: CI = D1/Dx1 + D2/Dx2, where Dx1 and Dx2 indicate the individual concentrations of ATO and AT7519 required to inhibit a given level of viability index and D1 and D2 are the concentrations of ATO and AT7519 necessary to produce the same effect in combination, respectively. The experiment process has been described in detail in our previous article (16).

*Acridine orange staining assay*

To investigate the contributory role of autophagy in AT7519 anti-leukemic effects on KG-1, cells were treated with an autophagy inhibitor CQ (40 µM) and then washed for three times with PBS. One μg/mL acridine orange (Merck, Darmstadt, Germany) was then added to each sample and after remaining for 15 min in the dark, the differences in acidity of autophagic lysosomes and cytoplasm/nucleolus were visualized under a fluorescence microscope (Labomed, Los Angeles).

*Statistical analysis*

Data were expressed as the mean ± standard deviation (SD) of three independent experiments. All experiments were performed in triplicate. All presented data were analyzed using GraphPad Prism Software using two-tailed student’s test and one-way variance analysis. In order to compare between the control group and the experimental ones, the Dennett’s multiple comparison test was used. A probability level of *P* < 0.05 was considered statistically significant.

## Results

*CDK inhibition using AT7519 disturbed cell cycle in AML-derived KG-1 cells*

**Figure 1:**
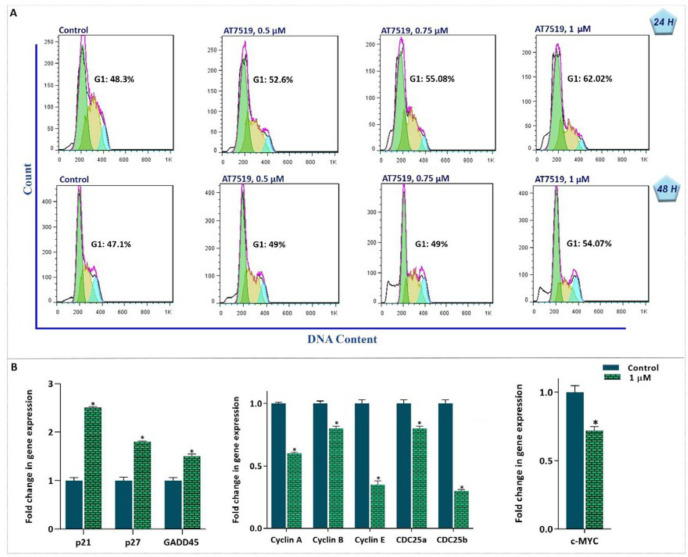
AT7519-induced G1 arrest is coupled with alteration in expression of the cell cycle-associated genes. (A) Evaluating the effect of the agent on the distribution of cells in different phases of the cell cycle revealed that AT7519 blocked the transition of the cells from G1 phase along with reduced the percentage of cells in S phase of the cell cycle. (B) The inhibitor altered the mRNA expression level of the cell cycle related genes. Values are given as mean ± SD of three independent experiments. ^*^P ≤ 0.05 represents significant changes from untreated control

Recent studies have indicated that treating cancer cells with cyclin-dependent kinase (CDK) inhibitors prevented cell cycle progression and accumulates cells in different phases of the cell cycle, mostly via down/up regulating the important proteins controlling cell proliferation (17). To investigate the effect of the CDK inhibition in acute leukemia, AML-derived KG-1 cells were treated with different concentrations of small molecule inhibitor of CDK AT7519 and then, the distribution of cells in different phases of cell cycle was examined using PI staining after 24 h and 48 h. As shown in Figure 1A, not only AT7519 dysregulated cell cycle progression and increased the number of KG-1 cells in G1 phase in a concentration-dependent manner, but also reduced the cell proportion in S phase. Of note, the results of qRT-PCR analysis indicated that the effect of AT7519 on the progression of cell cycle was coupled with the elevation in the expression levels of p21 and p27 together with the reduction in the mRNA levels of different cell cycle-associated cyclins (Figure 1B). It has been reported that through interaction with c-Myc and CDC25, GADD45 in connection with p21 could provide a signal leading to cell cycle arrest at both G1/S and G2/M transitions (18). In agreement with the results of p21, the mRNA level of GADD45 raised steeply in a concentration-dependent manner, while the expression level of CDC25 (α and β) reduced significantly upon CDK inhibition; however, evaluating the c-Myc mRNA level revealed that there is a slight reduction in the expression of this oncogene in response to AT7519 treatment (Figure 1B). 

*Anti-leukemic effect of AT7519 in KG-1 was enhanced upon PI3K and c-Myc inhibition*

**Figure 2:**
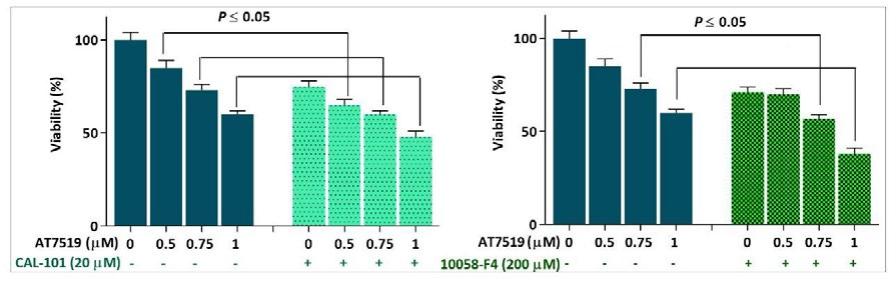
Suppression of the PI3K and c-Myc in KG-1 cells potentiated the anti-leukemic effect of AT7519. Values are given as mean ± SD of three independent experiments. ^*^P ≤ 0.05 represents significant changes from untreated control

Given to the minimal effect of AT7519 on c-Myc expression and on the basis of the role of c-Myc in bypassing the anti-survival signals induced by anti-cancer agents (19), it was of particular interest to evaluate c-Myc interference on the anti-leukemic effect of AT7519 in KG-1 cells. Notably, when we suppressed PI3K-mediated activation of c-Myc using isoform-specific P110δ inhibitor CAL-101, both the survival and proliferative rate of AT7519-treated KG-1 cells were reduced more vigorously as compared with either agent alone (Figure 2). Our finding was further strengthen by the results obtained from direct inhibition of this oncoprotein using small molecule inhibitor of c-Myc 10058-F4. In agreement with the inhibition of PI3K, we found that the combination of 10058-F4 with AT7519 resulted in a superior cytotoxicity in KG-1 cells (Figure 2); suggesting that either direct or indirect suppression of c-Myc could be a profitable strategy to boost the anti-cancer effect of CDK inhibitors in acute leukemia.

*Anti-survival effect of AT7519 either as a single agent or in combination with arsenic trioxide on KG-1 cells*

**Figure 3:**
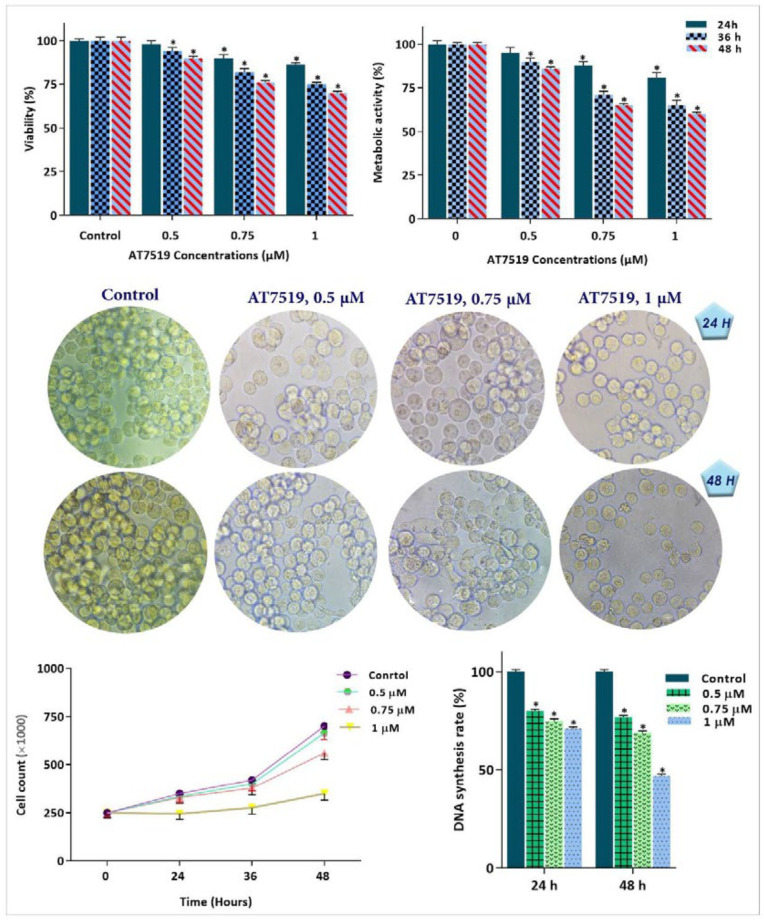
Cytotoxic effect of the CDK inhibitor on KG-1 cells. CDK suppression using AT7519 not only reduced the survival and proliferative capacity of KG-1 cells, but also restrained the DNA synthesis rate and diminished the number of viable cells. Values are given as mean ± SD of three independent experiments. ^*^P ≤ 0.05 represents significant changes from untreated control

**Figure 4:**
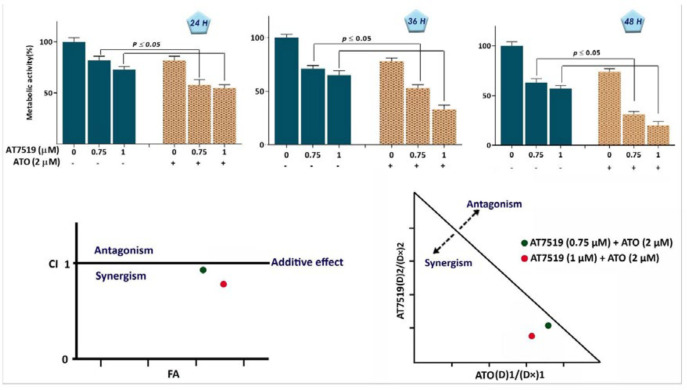
AT7519 and arsenic trioxide (ATO) co-treatment resulted in a superior cytotoxicity in KG-1 cells. (A) AT7519 could amplify the anti-cancer impact of ATO on KG-1 cells. (B) The results of both combination index (CI) and isobologram pointed out the synergistic effect between AT7519 and ATO. Values are given as mean ± SD of three independent experiments. ^*^P ≤ 0.05 represents significant changes from untreated control

To investigate whether the inhibitory impact of AT7519 on CDKs is associated with the anti-survival and anti-proliferative effects on KG-1, cells were treated with different concentrations of AT7519 and then, were subjected to trypan blue, MTT and BrdU assays. As expected, the anti-leukemic effect of the inhibitor was coupled with the reduction of cell survival, as revealed by the remarkable decreased in cell growth, metabolic activity and proliferative capacity of KG-1 cells (Figure 3). Intrigued by the remarkable anti-tumor effect of AT7519, we also aimed to determine whether this agent could enhance the cytotoxic effect of arsenic trioxide (ATO), a common chemotherapeutic drug used in AML treatment. As shown in Figure 4, the combination of AT7519 with ATO (2 µM) was more effective in inhibiting the cell growth and survival as compared with either drug alone. Combination index (CI) and dose reduction index (DRI) further confirmed the stimulatory effect of CDK inhibition on ATO cytotoxicity in AML-derived KG-1 cells (Table 1), and highlighted that AT7519, either alone or in combination with chemotherapeutic drugs, is a promising agent in the treatment strategy of acute leukemia. 

*AT7519-induced cytotoxicity was mediated through apoptotic pathway*

**Figure 5:**
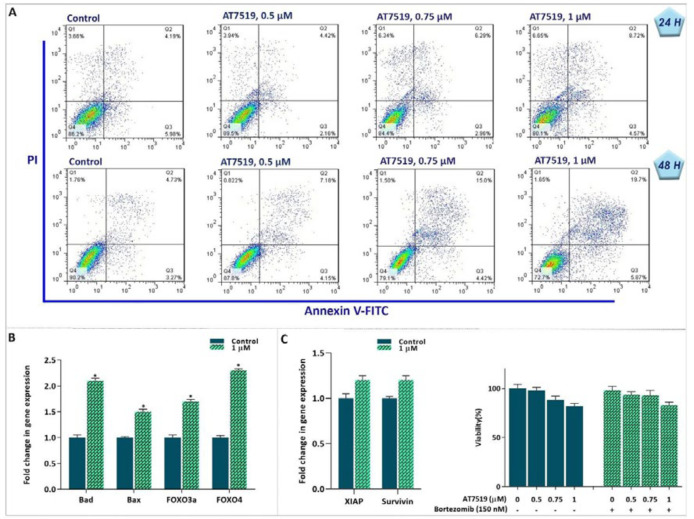
Suppression of CDK in KG-1 cells was coupled with induction of apoptotic cell death. (A) AT7519 increased the percentages of Annexin-V and Annexin-V/PI double positive cells in KG-1 cells. (B and C) After treatment of the cells with AT7519, the expression levels of apoptotic genes were determined using qRT‐PCR. Although the inhibitor could increase the expression of pro-apoptotic genes, it failed to impede the expression levels of anti-apoptotic genes. In agreement, the proteasome inhibitor was not capable to boost AT7519 cytotoxic effect. * *P* ≤ 0.05 represents significant changes from untreated control

Mounting body of evidence has declared that in addition to their role in the progression of the cell cycle, CDKs could also control the death signal in malignant cells (20). To determine whether the cytotoxic effects induced in inhibitor-treated cells were likely because of the induction of apoptosis, the binding of annexin-V combined with PI were analyzed by flow cytometry. We found that there is a considerable increase in both annexin-V and annexin-V/PI double-positive cells after 24 h and 48 h exposing the cells to AT7519 (Figure 5A). To gain more insight into the molecular mechanisms responsible for the induction of apoptosis, we scrutinized the mRNA expression levels of pro- as well as anti-apoptotic-related genes. The results of qRT-PCR showed that while AT7519 increased the expression of Bax, Bad, Foxo3a and Foxo4a, this agent failed to hamper the expression levels of anti-apoptotic genes (Figure 5B). It was suggested that the activation of the proteasome pathway upon drug treatment could directly seize the induction of apoptosis through neutralizing the degeneration of anti-apoptotic proteins (21). However, when we treated KG-1 cells with a well-known proteasome inhibitor bortezomib in combination with AT7519, we failed to find any significant enhancive effect on the viability and survival capacity of cells (Figure 5C); suggesting that the cytotoxic effect of the CDK inhibitor was not probably influenced by the activation of the proteasome pathway.

*AT7519 synergized with autophagy inhibitor CQ to augment cytotoxicity in AML cells*

**Figure 6:**
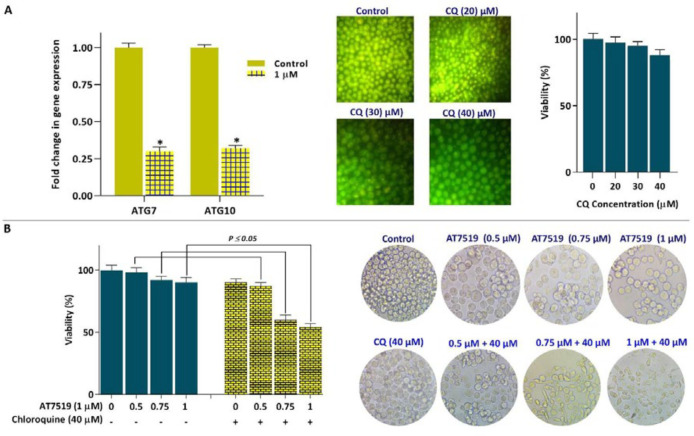
Co-treatment of AT7519 and autophagy inhibitor CQ led to superior cytotoxicity in KG-1 cells. (A) Inhibition of CDK using AT7519 down-regulated the mRNA expressions of both ATG7 and ATG10 in KG-1 cells. (B) CQ reinforced the anti-cancer impact of AT7519 in KG-1 cells. Values are given as mean ± SD of three independent experiments. ^*^P ≤ 0.05 represents significant changes from untreated control

A growing body of evidence demonstrated that the majority of CDKs and CKIs could affect the fate of cancer cells through the modulation of autophagy system (22). Given this, we were interested in examine whether AT7519 could alter the mRNA expression of autophagy-related genes in KG-1 cells. The resulting data showed that the inhibition of CDK using AT7519 down regulated the mRNA expressions of both ATG7 and ATG10 (Figure 6A). The association between autophagy and cell death has been examined in different cancer cell types; however, in many cases there are conflicting results. While some reports introduced autophagy system as mechanism through which anti-cancer agents induced cell death in cancer cells, other studies referred this system as a resistance mechanism against drugs (23). To investigate whether the inhibition of autophagy-related genes expression acts as a protection or an execution mechanism of cell death, KG-1 cells were also treated with an inhibitor of autophagy chloroquine (CQ). The resulting data showed that not only the autophagy inhibition, as revealed by the decreased excitation of acridin florescent, decreased the viability of inhibitor-treated KG-1 cells (Figure 6B), but also simultaneous suppression of both autophagy and CDK resulted in a superior anti-leukemic effect on KG-1 cells; shedding light that the inhibition of autophagy in AT7519-treated cells may probably serve as an executioner of cell death.

**Figure 7:**
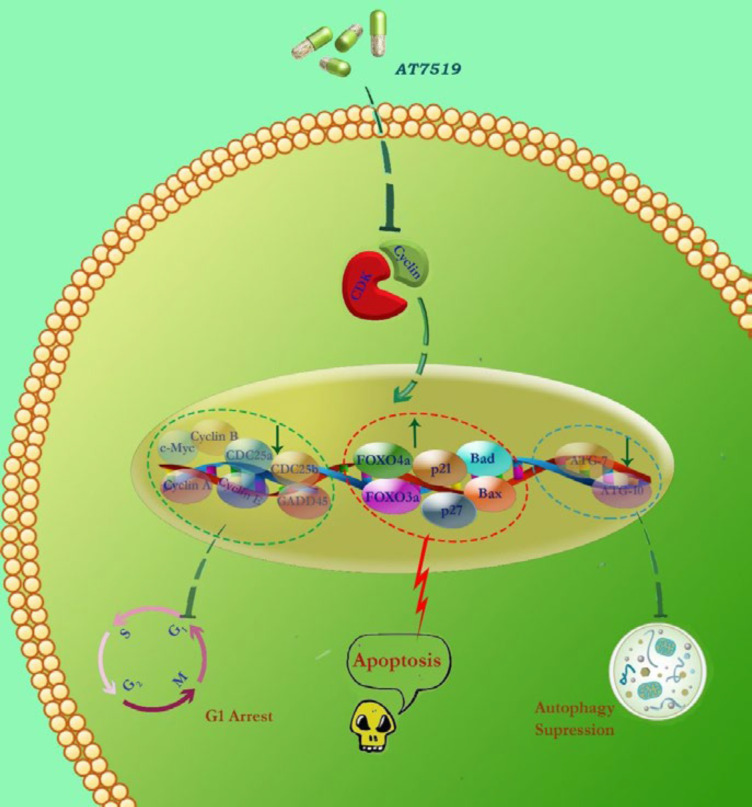
Schematic representation proposed for the presumable mechanisms of action of AT7519 in KG-1 cells. Via abrogation of the CDK and induction of G1 cell cycle arrest, AT7519 diminished the survival and proliferative capacity of the cells plausibly through altering both cell cycle- and apoptotic-related genes. We found that the blockage of autophagy system in KG-1 cells resulted in a superior cytotoxic effect; introducing autophagy as a probable suppressor of cell death

**Table 1:** Combination index (CI) and dose reduction index (DRI) for drug combination by AT7519 and ATO

**AT7519**		**ATO**		**CI**
**Concentration (μM)**	**DRI**	**Concentration (µM)**	**DRI**	
0.75	4.62	2	1.39	0.93
1	6.79	2	1.58	0.77

## Discussion

On the basis of the recent medical advances, novel molecular findings have changed the face of the traditional therapeutic strategies by introducing susceptible targets to better attack the malignant cells. The identification of the cyclin-dependent kinase (CDK) inhibitor proteins and further comprehension of their roles in the regulation of diverse intracellular functions have sparkled the idea of the advent of CDK inhibitors into the cancer treatment protocols (17). In the present study, we examined restrictive potential of pan-CDK inhibitor AT7519 on the cellular and molecular features of acute myeloid leukemia-derived KG-1 cells. Our data showed that the abrogation of CDKs, as revealed by the induction of G1 cell cycle arrest coupled with a significant reduction in the expression of cyclins, resulted in decreased survival in KG-1 cells either in context of single agent or in combination with arsenic trioxide (ATO). Once the transition of cells from G1 phase of the cell cycle is blocked, the first regulator that assumed its expression would be changed is GADD45, a stress sensor with multiple connections with different intracellular proteins (18). Accordingly, FOXO family members, which are engaged in a unique crosstalk not only with the cell proliferation but also with the induction of apoptotic cell death, have been suggested as one of the most important molecules having an associated with GADD45 (24). Previous studies have also declared that the missing chain for FOXO family to orchestrate both cell proliferation and cell death is p21 cyclin-dependent kinase inhibitor which could promote apoptosis through cleavage of caspase-3 (25, 26). Interestingly, our results clearly showed that the suppression of CDK in KG-1 cells increased the expression of p21 through elevating the expression of GADD45. Moreover, this agent was able to induce apoptotic cell death in KG-1 cells by increasing the expression levels of FOXO-related apoptotic genes. 

For a long time, the biological role of c-Myc in malignant cells has been tightening to the regulation of the cell cycle; however, novel molecular investigations have enumerated other functions for this oncogene (27). It has been suggested that c-Myc plays a key role in the blockage of death stimuli in neoplastic cells, increasing the metabolic activity and disturbing crucial signaling pathways (28, 29). Additionally, c-Myc is now considered as prognostic marker in many leukemic patients, since its overexpression leads to tumor recurrence and relapse (30, 31). Although c-Myc is involved in cell cycle progression and has an association with GADD45 (32), our results showed that AT7519 failed to significantly suppress its expression in KG-1 cells. There are some reports suggesting that the PI3K signaling pathway, which is activated in KG-1 cells to maintain its stemness feature, is a leading cause of c-Myc overexpression in leukemic cells (33, 34). Collaborative with these reports, when we blocked the expression of c-Myc either by directly suppressing c-Myc or by inhibiting the PI3K pathway, the anti-leukemic effect of AT7519 was ameliorated more significantly; indicating that the effectiveness of AT7519 could be overshadowed, at least partly, by c-Myc and PI3K activation. In consistent, the critical roles of both c-Myc and PI3K pathways in determining the sensitivity extent of cancer cells to the anti-cancer agents have been reported in our recent investigations, indicating that the suppression of these onco-proteines may be a beneficial approach in combined-modal strategies (35-39). 

The correlation between CDKs and autophagy has long been under intense examination, introducing a dilemma whether targeting autophagy could be profitable for cancer therapy (40). While there is a consensus to regard a unique tumor suppressor mode for autophagy, others declared that the exploiting autophagy inhibitors alongside chemotherapeutics drugs reinforce their efficacy (41). In this regard, it has been indicated that the inhibition of autophagy in leukemic cells could enhance the anti-leukemic effects of some small molecule inhibitors of oncogenic pathways (42, 43). Interestingly, apart from alterations in the expression of the genes involved in cell proliferation and apoptosis, we found that the suppression of CDK was coupled with the inhibition of autophagy-related genes. Notably, our result was further confirmed in a combinatorial experiment where we found that the anti-leukemic effect of AT7519 was enhanced upon pharmacologic inhibition of autophagy using chloroquine; shedding light that the inhibition of autophagy in AT7519-treated cells may probably serve as an executioner of cell death. In agreement, there are some other investigations reporting that the suppression of autophagy may provide a platform for anti-cancer agents to more vigorously induce their anti-survival property (35, 43). By mentioning a proportion of studies reporting controversial findings about the amplifying role of autophagy inhibitors, it may be reasonable to conclude that the origin of the tumor cells and the drug combination could affect the anti-cancer property of autophagy inhibitors (Figure 7) (40). Overall, this study suggests that AT7519 is a promising anticancer agent, either as a single agent or in a combined-modality strategy; however, further investigation should be accomplished to determine the usefulness of the inhibitor in cancer therapeutics, in particular for the treatment of acute myeloid leukemia.
